# 

**Published:** 1888-03-03

**Authors:** 


### SPECIAL NOTICE TO NEWSAGENTS, ADVERTISERS, AND THE TRADE.

### CHANGE OF OFFICE AND PUBLISHER.

Notice is hereby given that the Office of The Hospital will henceforth be at 38, Old Jewry (Cheapside end), E.C., to which address all communications should be sent. Mr. A. Doig has been appointed Advertising Agent, and Mr. J. H. S. Hanning, Manager. All Cheques and Post Office Orders should be made payable to J. H. S. Hanning, 38, Old Jewry, E.C. crossed Lloyds, Barnetts, and Bosanquets Bank (Limited).

### Amusements with Profit for all Ages.

**Rules.**—All answers must be addressed to the Prize Editor, The Hospital, 38, Old Jewry, Cheapside, London, E.C., not later than the *first* post on Thursday next. The full name and address must in all cases be given, but a *nom de plume* may be added for publication. Only one side of the paper must be written on.

### FOR MEN AND WOMEN.

**Rule.**—Competitors can enter for *all* quarterly competitions, but no competitor may take more than *two quarterly prizes* during the year.

### Acrostic Competition.

Second quarter commences February 25th, 1888; ends May 19th, 1888.

A PRIZE OF ONE GUINEA will be given to the competitor who gains the highest number of marks for best original acrostics during the ensuing quarter. All acrostics sent in for this competition will be credited according to merit, twelve marks being the highest score given for one acrostic.

A PRIZE of HALF-A-GUINEA to be given to the competitor who gains the highest score for solution of acrostics set. *One mark* only will be given to the competitors who solve all the lights of each acrostic.

Exception will be made in the case of *quotation acrostics*, when *two marks* will be given, *one* for the correct solution of all *lights*, and *one* mark for the *source* of the quotations.

This week credit will be given for

**No. 1 Original Acrostic.**

### Answers.

No. 7. Double Acrostic.

| | | |
|---|---|---|
| I | n | K |
| G | ilpi | N |
| N | egr | O |
| O | utla | W |
| R | ache | L |
| A | ndr | E |
| N | o | D |
| C | lin | G |
| E | vangelin | E |

Correct answers have been received from Tabs, Gretta, Ellice, E. E., Brisbane, Condorcet, Dunce, Reldas, Lady Clara, Patience, Mater, Tom Cobb.

Results of Original Acrostic Competition.—*Brownie* and *Pasteur* having each gained the highest score of marks in the above competition, we give them the option of dividing the prize of *One Guinea*, or of composing a *Test Acrostic*, the subject for the same *to be set by the Prize Editor*.

Results of Set Acrostic Competition.—The above competition has resulted in a tie between *Condorcet* and *Dunce*, who have each sent in the highest number (6) correct solutions. We shall be glad to hear from them, whether they prefer to divide the prize, or would like another acrostic to be set for final solution.

### (A) THE WORD COMPETITION.

Second Quarterly Competition commenced February 4th, 1888; ends April 28th, 1888.

Three prizes of one guinea, 15*s.* and 10*s.* 6*d.* will be given each quarter to the three competitors who obtain the highest number of marks; (1) by making the largest number of words derived from the word or phrase set for anatomical dissection; or (2) for the best answers to (B); or (3) by (1) and (2) combined.

We shall give the newspapers and periodicals for dissection during this quarter, that for this, the *fifth* week of the quarter, being

"The Echo."

Observe.—Proper names, abbreviations, foreign words, words of less than four letters, and repetitions are barred; plurals, past and present participles, or verbs are allowed. Nuttall's *Standard* dictionary only to be used.

N.B.—All words sent in must be arranged *alphabetically*, with correct total affixed.

### Results of Third Week.

*The World.*—Ellice (96), Patience (95), Gretta (91), Dollie (8?), Isabel (80), Lodge (77), E. E. (77), Susan Nipper (73), Lauriston (72), Lightowlers (70), Ninny (69), Rowdy Corner (68), Lancashire Witch (68), Brisbane (67), Sym (61), Dunce (61), Condorcet (61), Nelle (56).

### (B) THE LITERARY COMPETITION.

Some people hate puzzles of all kinds, including word competitions, but they are fond of correspondence, and many sisters and mothers are continually receiving and sending letters to and from members of the family who are abroad in India or the Colonies. The constant coming and going between England and other portions of the British Empire make it a matter of interest and importance to know something about the life, the climate, the amusements, the adventures, the clothing, and the surroundings of those who seek a livelihood beyond the seas. We shall therefore try to find space for the publication of selected letters from abroad, which may be sent by any of our readers who have friends and relations in foreign lands. We shall give marks for these letters as well as for the word competitions which will count equally for these prizes, so that anyone may enter for both or either, the prizes to be given to those who get the highest number of marks.

### Letters from Across the Sea.

*May* sends the following letter from New York and Toronto.

New York.

Here I am again after nine months' absence, feeling all the better for my delightful holiday in my native land. The first thing that struck me on landing was the clearness of the atmosphere, the blueness of the skies, and the magnificence of the buildings. Dear old smoky London was quite put in the shade. I put up at one of the hotels I am accustomed to go to. It used to be first-class, but now it is a second-rate one; however, it is magnificent. Three hundred persons sat down to each meal, and the ménu was capital, so much more varied than in English hotels. I had a miserable voyage out. As I wrote to you from Queenstown, owing to the accident to our vessel, *The City of Richmond*, we—that is to say, all the passengers—were transferred from our own vessel to another, a wretched one with not nearly enough room for the numerous passengers. Contrary to my expectations, the weather was very rough and stormy. However, I was not ill. One fact struck me very forcibly. When I came over in March the vessel was then full of American and Canadian produce. In returning we brought with us British gold instead; this cannot be for the good of England. To-morrow I start off for Toronto and work.

Toronto.

Last week I took a trip to Montreal by boat, had a state-room to myself, excellent meals, and magnificent weather. The boat was one that the Prince of Wales used in his Canadian trip, a splendid smooth sailer. You have little idea how magnificently these boats are fitted up. In the evening you dress, go into the saloon, play chess, draughts, bagatelle, and listen to the singing and music of the lady passengers. There was one fellow who recited splendidly for our amusement. On our way we stayed about two hours at Kingston, a beautifully situated town with lovely surrounding scenery; houses and public buildings built of stone. From Kingston the river is exceedingly wide, varying from three to seven miles: studded with islands, some covered with verdure, others barren rocks. Where the river is widest it goes by the name of the "Lake of a Thousand Isles." The scenery is lovely, though they say that from Montreal to Quebec it is very much better. Where the river is narrow, rapids form, and much care has to be taken in navigating, as the current is very strong. We stayed at Lachine early in the morning, and arrived at Montreal an hour after. The first attraction was the Victoria Bridge, which in the distance looked like a line drawn across the river; but as we approached nearer the magnificent proportions of the structure filled us with admiration. It is nearly two miles across. The next attraction was the two towers of Notre Dame Cathedral. When close to the city I felt rather disappointed, but soon lost the feeling in curiosity. Everything seemed foreign: people talking French, French style of buildings, priests, nuns, sisters of mercy everywhere. Some of the most magnificent buildings are nunneries, and Roman Catholic schools accommodating three or four hundred inmates. Some of the streets are very old and narrow. The name Montreal is taken from the mountain which forms the background of the city, Mount Royal. This is about 800 or 900 feet in height. I had a splendid drive all round this mountain, about nine miles. The view across the river was magnificent, with the bridge, steeples, and towers of the city below. On the opposite side of the river is another mountain, the end of a range stretching away to Vermont. My trip altogether was most interesting. I send you photographs of the principal places I saw. I shall hope to visit the city again, and will then tell you more.

### THE CHILDREN'S CORNER.

PRIZES.
FOR MOST CORRECT ANSWERS TO PUZZLES.

This Commenced October 1st, 1887.

One Pound a Month.

A PRIZE OF THREE GUINEAS to the Competitor who shall have sent in the largest number of correct or best answers during the twelve months.

### Puzzles—First Week.

No. 1. Double Acrostic.—1. A marine plant. 2. Black and musical. 3. A North American river. 4. Striped and venomous. Initials and finals give the name of a spring flower.

No. 2. Enigmatical List of Distinguished Persons.—1. A savage animal and a vowel. 2. A verb and a sharp weapon. 3. A useful animal and half an individual. 4. A kind of clay and a Corporation town.

No. 3. Charade.

My *first* makes all nature appear with one face;
My *second* has music and beauty and grace;
And if this charade is not easily known,
My *whole* at your head you deserve to have thrown.

No. 4. A duck before two ducks, a duck behind two ducks, and a duck between two ducks. How many ducks were there in all.

### Results—Third Week.

No. 9. Enigma. Potatoes.

No. 10. Geographical Diamond Puzzle.

```
        D
      A A R
    F A R O E
  G R I M S B Y
D A R M S T A D T
  P O R T S E A
    S P A I N
      I D A
        T
```

No. 11. Bees are wise insects.

No. 12. Query. Alone, Lone, One.

*All right*—Alert, A. G. S., McCulloch, A. C. K., Squeaker, Scrooge, Excelsior, Oswald, H. Robinson. *Three right*—Mount Edgcumbe, Jerusalem, Plummy Fluff, Gem, Spider. *Two right*—Moina, Alsie, A. T., Happy Eliza, Judy.

E. M. Warner is referred to Rule 4 of the conditions of these competitions, which appeared in the first number of "The Hospital," and have been published from time to time as space permitted. She was bound, like all other competitors, by these conditions, and her contentions prove the necessity of enacting that the prize editor's decision is in all cases final and without appeal. After investigating the matter, we cannot refrain from expressing surprise and regret that such a claim should have been advanced at all, as it is wholly unjustifiable, and ought never to have been made

### Conditions.

I. Every paper sent in must be clearly written, and one side only must be used. All competitors must mark at the head of their papers the number of their competition.

II. Each paper must be signed by the author with his or her real name and address. A *nom de plume* may be added, if the writer does not desire to be referred to by us by his real name. In the case of all prize-winners, however, the real name and address will be published.

III. All communications relating to prize competitions should be addressed to "The Prize Editor, The Hospital, 38, Old Jewry, London, E.C." Competitors are requested not to include in letters, etc., to the Prize Editor communications on matters of other business. All letters must be fully prepaid.

IV. The Prize Editor of "The Hospital" is the sole judge of the competitions, and his decision is in all cases final and without appeal.

V. The Prize Editor reserves the right to publish any paper sent in, whether it receives a prize or not. He does not hold himself responsible for any MSS. sent, nor can he undertake to return rejected competitions.

**Notice to all Correspondents.**—N.B.—All letters referring to this page, which do not arrive at 38, Old Jewry, Cheapside, London, E.C., by the *first post on Thursdays*, and are not addressed PRIZE EDITOR, will in future be disqualified and disregarded.

No one will be permitted to win the Monthly or Quarterly Prizes twice in any one year, the annual prizes being offered especially to prevent this.

